# Entwicklungen der Österreichische Gesellschaft für Kinder- und Jugendpsychiatrie, Psychosomatik und Psychotherapie – Ergebnisse einer Mitgliederbefragung

**DOI:** 10.1007/s40211-020-00381-7

**Published:** 2020-11-27

**Authors:** Leonhard Thun-Hohenstein, B. Ecker

**Affiliations:** 1 Österreichische Gesellschaft für Kinder- und Jugendpsychiatrie, Psychosomatik und Psychotherapie (ÖGKJP), Währinger Gürtel 18–20, 1090 Wien, Österreich; 2 Univ.-Klinik für Kinder- und Jugendpsychiatrie der SALK/CDK, Paracelsus Medizinische Privatuniversität Salzburg, Ignaz Harrerstraße 79, 5020 Salzburg, Österreich

**Keywords:** Non-Profit-Organisation, Organisationsentwicklung, Mitgliederbefragung, Kinder- und Jugendpsychiatrie, ÖGKJP, Non-profit organisation, Organisational development, Member survey, Child and adolescent psychiatry, ÖGKJP

## Abstract

**Hintergrund:**

Die Fachgesellschaft ÖGKJP ist – bedingt durch die gesetzlichen Änderung 2015 – in einem dynamischen Entwicklungsprozess. Der Vorstand hat in den vergangenen Jahren einen Organisationsentwicklungsprozess durchlaufen und neue Strukturen und strategische Zielsetzungen entwickelt. Die Etablierung dieser Strukturen und die Erreichung dieser Ziele sollen durch verschiedene Maßnahmen und Angebote sichergestellt werden. Ziel dieser Studie war es, über eine Mitgliederbefragung in Erfahrung zu bringen, wie wichtig die Mitglieder diese Maßnahmen und Angebote erachten und wie zufrieden sie damit sind. Weiters wurden die Befragten dazu eingeladen, bereits definierte Zukunftsthemen in Bezug auf ihre Wichtigkeit zu bewerten und weitere Themen, die in Zukunft von der Fachgesellschaft (intensiver) bearbeitet werden sollen, vorzuschlagen.

**Methodik:**

294 via E‑Mail erreichbare Mitglieder erhielten unter Heranziehung der Online-Umfrage-Applikation LimeSurvey einen von ÖGKJP-Vorstandsseite entwickelten Fragebogen. Der Fragebogen bestand aus 12 geschlossen Fragen, die entlang von 5 Themenblöcken (siehe weiter unten) sowohl die Wichtigkeit von Themen behandelten als auch die Zufriedenheit mit der bisherigen Arbeit der Fachgesellschaft. Die Bewertungen wurden entlang einer fünfteiligen Skala (1 = sehr wichtig/zufrieden, 2 = wichtig/zufrieden, 3 = moderat wichtig/zufrieden, 4 = wenig wichtig/zufrieden, 5 = gar nicht wichtig/zufrieden) erbeten. Die Auswertung erfolgte mittels deskriptiver Statistik.

**Ergebnisse:**

101 Mitglieder (knapp 35 %) haben den Fragebogen beantwortet. 65 % der Antwortgeber*innen waren ordentliche Mitlieder, 15 % ordentliche Mitlieder in Ausbildung und 8 % außerordentliche Mitglieder. 12 % erteilen keine Angaben zu ihrem Mitgliedsstatus.

Beim Thema *Forschung und Wissenschaft* bewerteten die Mitglieder die Wichtigkeit im Durchschnitt zwischen 1,53–2,55 und die Zufriedenheit mit der Umsetzung zwischen 1,50 und 2,16. Hinsichtlich des Themas *Veranstaltungen und Kooperationspartnerschaften* liegt die Wichtigkeit zwischen 1,63 und 2,10, die Zufriedenheit mit der Umsetzung zwischen 1,55 und 2,22. Die *Repräsentanz des Faches in der Öffentlichkeit* wurde als höchst wichtig erachtet (1,23), die Zufriedenheit in Bezug darauf liegt bei 2,24 Punkten. Die *Neuerungen im Servicebereich der Gesellschaft* wurden hinsichtlich ihrer Wichtigkeit wie folgt beurteilt: Homepage 1,82, Lernplattform Moodle 2,28 und das neue Sekretariat mit 1,83. Die Zufriedenheit der Mitglieder mit diesen Neuerungen bekommt jeweils die Note 2,22; 2,50 und 1,84.

**Diskussion:**

Bei zufriedenstellender Beteiligung wird der Fachgesellschaft eine gute, aber ausbaubare Zufriedenheit signalisiert. Die Wichtigkeit der vom Vorstand vorgenommenen Themenwahl wurde bestätigt. Zukunftsthemen, die von den Mitgliedern als besonders wichtig bewertet wurden, sind Veranstaltungen und Kooperationspartnerschaften, sowie die Repräsentanz der ÖGKJP auf politischer Ebene.

## Summary

**Background:**

Due to legal changes in 2015, the medical specialist society ÖGKJP is in a dynamic development process. In recent years, the Executive Board has gone through an organisational development process and developed new structures and strategies. The aim of this study was to ask about the importance of the various offers and objectives and the satisfaction with the events and measures via a member survey. Respondents were also invited to identify future topics and to evaluate their importance.

**Methods:**

249 members could be reached by email and had been provided with a self-developed questionnaire to be answered by an online-application (Lime-Survey). The questionnaire consisted of twelve closed questions for five topics (see result section). The question could be answered by a Likert-like-scale (1 very content to 5 not at all content). Statistical analysis was performed by descriptive tests.

**Results:**

101 members (nearly 35%) have replied to the questionnaire. 65% of the responders were regular members, 15% were regular members in training and 8% were extraordinary members. 12% do not give any information about their membership status

With regard to the topic *research and science*, importance was rated on average between 1.53 and 2.55 and satisfaction with the implementation between 1.50 and 2.16. With regard to the topic *events and cooperation partnerships*, the importance was rated between 1.63 and 2.10, the satisfaction with the implementation between 1.55 and 2.22. The *representation of the organisation in the public* was considered to be highly important (1.23), the satisfaction of the members is 2.24 points. The innovations in the *service of the company* were assessed in terms of their importance as follows: homepage 1.82, learning platform Moodle 2.28 and the new secretariat with 1.83. The satisfaction of the members with these innovations is given the grades 2.22, 2.50 and 1.84.

**Discussion:**

Participation was good, members rated satisfaction on a good, but improvable level. Importance of selected topics was confirmed and two future topics were pointed out especially, events and cooperation partnerships as well as the representation of the ÖGKJP at the political level.

## Einleitung

Das Sonderfach Kinder- und Jugendpsychiatrie und Psychotherapeutische Medizin besteht in Österreich nun seit 2015. Die Vorläufer dieses Faches waren das Zusatzfach für Kinder- und Jugendneuropsychiatrie seit 1975 und das Sonderfach Kinder- und Jugendpsychiatrie ab 2007. Letztere Einführung hatte ganz wesentliche Auswirkungen auf die Versorgungslandschaft der Kinder- und Jugendpsychiatrie in Österreich, ermöglichte es doch die Aufnahme des Sonderfaches Kinder- und Jugendpsychiatrie in den österreichischen und regionalen *Strukturplan Gesundheit* und in Folge unter anderem die Schaffung neuer Krankenhausabteilungen und den – wenn auch langsamen – Ausbau des extramuralen Sektors.

Um die fachliche und politische Aufbauarbeit zu leisten, wurde 1975 die „Österreichische Gesellschaft für Kinder- und Jugendneuropsychiatrie“ gegründet. Diese wurde 2007 in „Österreichische Gesellschaft für Kinder- und Jugendpsychiatrie“ und 2015 in „Österreichische Gesellschaft für Kinder- und Jugendpsychiatrie, Psychosomatik und Psychotherapie“ umbenannt [1]. Durch die Einführung des Sonderfaches, insbesondere durch die Österreichische Ärzteausbildungsordnung (ÖAO 2015 [2]), wurde auch die Ausbildung der Ärzt*innen durch die Integration der Ausbildung in Psychotherapeutischer Medizin nachhaltig verändert. Für die Fachgesellschaft bedeuteten diese Entwicklungen und die deutliche Zunahme der Mitglieder von >40 % die Notwendigkeit einer neuen strategischen Ausrichtung.

Die ÖGKJP ist ein Verein nach dem Österreichischen Vereinsgesetz [2] und als solcher eine Non-Profit-Organisation. In den letzten Jahrzehnten hat sich das Management von Vereinen professionalisiert, wenngleich das Management eines Vereins sich deutlich von klassischen Managementstrategien unterscheidet: Vereine sind häufig ideeller Natur und werden über Mitgliederbeiträge oder Spenden finanziert, verfügen über freiwillige und ehrenamtliche Mitarbeiter*innen und versuchen in der Regel demokratisch und partizipativ die Mitglieder in die Steuerung des Vereines einzubeziehen [3]. In der Weiterentwicklung unseres Vereins haben wir beim *Konzept der Wissensspirale* Anleihe genommen. Das Modell der Wissensspirale nach Nonaka und Takeuchi [4] beschreibt die Schaffung von neuem Wissen innerhalb eines Unternehmens. Die Hauptaufgabe von Wissensmanagement liegt darin, anderen Mitarbeiter*innen eines Unternehmens Wissen zur Verfügung zu stellen, das für die Bewältigung bestimmter Problemstellungen erforderlich ist. Dazu muss Zugang zu dem individuellen Wissen einer Person hergestellt werden und gleichzeitig soll neues Wissen entwickelt werden. Dieses entsteht durch einen Spiralvorgang (Wissensspirale) aus Mobilisierung und Transformation des bestehenden Wissens.

In den Jahren 2016–2018 hat sich der Vorstand mehrere Klausuren verordnet und eine neue strategische Ausrichtung der Fachgesellschaft erarbeitet. Gleichzeitig wurden auch die Strukturen der Gesellschaft neu geordnet und die Zielsetzungen geschärft. Die Ergebnisse dieser Strategieentwicklung wurden den Mitgliedern jeweils in den Generalversammlungen mündlich und schriftlich mitgeteilt [5].

Die Hauptergebnisse der strategischen Ausrichtung umfassten einerseits einen Neuaufbau der Selbstorganisation und eine Verbesserung der Servicierung der Mitglieder. In diesem Zusammenhang wurde ein fixes Sekretariat aufgebaut und die Homepage überarbeitet. Zudem wurde ein interner Mitgliederbereich bzw. eine Lernplattform (ÖGKJP Campus/Moodle) für die ÖGKJP-Akademie geschaffen.

Weiters erarbeitete der Vorstand sechs Strategien für die weitere Ausrichtung der Fachgesellschaft: Strategie Nummer 1 umfasst das Thema *Versorgung* mit dem Ziel, den Ausbau der kinderpsychiatrischen Versorgung im Sinne der Vollversorgung und die Errichtung spezialisierter Versorgungsangebote voranzutreiben. Strategie Nummer 2 betrifft die *Aus- und Weiterbildung* mit dem Ziel, die Ausbildung der Assistenzärzt*innen zu unterstützen und zu begleiten, gemeinsame Standards für alle Abteilungen sowie gemeinsame, entwicklungsbegleitende Qualitätskontrollen (Rasterzeugnis, Logbuch etc.) zu entwickeln, und schlussendlich die entsprechende Qualifikationskontrolle gemeinsam mit der Österreichischen Ärztekammer (ÖÄK) durch die Facharztprüfung durchzuführen. Auch den lebendigen fachlichen Diskurs unter den Fachärzt*innen zu initiieren und aufrechtzuerhalten und entsprechende Veranstaltungen und Publikationen anzubieten, ist wichtiger Teil dieser Strategie. Um diese Strategie bestmöglich zu unterstützen wurde die *ÖGKJP-Akademie* gegründet, die sich aus dem Curriculum CuPsy (Ausbildung Psychotherapeutische Medizin), den Assistent*innentagen und der Facharztweiterbildung (*Facharztwerkstatt Gösing*) zusammensetzt. Verantwortlich für diese neue Akademie ist die Ausbildungskommission.

*Wissenschaft und Weiterentwicklung* (Strategie Nummer 3) des Faches ist ein weiteres zentrales Thema der neuen strategischen Ausrichtung. Es geht um die Förderung von Wissenschaft und Forschung und die Weiterentwicklung aller Themen das Sonderfach betreffend auf wissenschaftlicher Basis. Zentral – auch im Sinne der Akquise von neuen Assistenzärzt*innen – ist der Abgleich und Ausbau der universitären Lehre im Rahmen des Medizinstudiums.

Durch die Vergrößerung und die zunehmende politische Bedeutung der Fachgesellschaft wird es zwingend notwendig auch *Öffentlichkeitsarbeit nach innen und nach außen* (Strategie Nummer 4 und 5) zu betreiben. Es gilt die Außenwirkung der Fachgesellschaft, und vor allem der Situation der Kinder- und Jugendpsychiatrie, in der Öffentlichkeit darzustellen und weiter voranzutreiben, aber auch nach innen die Mitglieder über Erfolge, Tätigkeiten, Ergebnisse der Arbeit der Fachgesellschaft rasch und effizient zu informieren. Die Strategie Nummer 6 *Kooperation* umfasst die Verbesserung der kontinuierlichen Zusammenarbeit mit relevanten Partnern und Stakeholdern im medizinischen gesellschaftlichen, aber vor allem auch im politischen Bereich.

Um im Sinne der lernenden Organisation auch die potenziellen Nutznießer*innen der Arbeit der Fachgesellschaft zu integrieren, wurde eine Mitgliederbefragung beschlossen und Anfang des Jahres 2020 durchgeführt. Das zentrale Anliegen dieser Befragung war, ein breites Feedback zur Wichtigkeit und zur Zufriedenheit mit den bestehenden Angeboten und Services der ÖGKJP zu erhalten, sowie Anregungen und Ideen für die weitere Entwicklung der Fachgesellschaft von Seiten der Mitglieder zu bekommen.

## Methode

Die Vorbereitungsarbeiten und die Erstellung des Fragebogens erfolgten Seitens des ÖGKJP-Vorstandes im Herbst 2019. Umgesetzt wurde die Befragung zwischen dem 15. Jänner und 15. Februar 2020 unter Heranziehung der Online-Umfrage-Applikation LimeSurvey.

Zur Teilnahme an der Befragung eingeladen wurden die damals 294 (via E‑Mail erreichbaren) der insgesamt 300 ÖGKJP-Mitglieder. 101 Mitglieder (knapp 35 %) haben Antworten erteilt. Eine Unterscheidung entlang von Mitgliederkategorien zeigt, dass in etwa 65 % der Antwortgeber*innen ordentliche Mitlieder waren, 15 % ordentliche Mitlieder in Ausbildung und 8 % außerordentliche Mitglieder. 12 % erteilen keine Angaben zu ihrem Mitgliedsstatus.

## Der Fragebogen

Die Befragung bestand aus 12 geschlossen Fragen, die entlang von 5 Themenblöcken (siehe weiter unten) sowohl die Wichtigkeit des Themas erfragten als auch die Zufriedenheit mit der bisherigen Arbeit der Fachgesellschaft. Die Bewertungen wurden entlang einer fünfteiligen Skala (1 = sehr wichtig/zufrieden, 2 = wichtig/zufrieden, 3 = moderat wichtig/zufrieden, 4 = wenig wichtig/zufrieden, 5 = gar nicht wichtig/zufrieden) erbeten.

### Themenblöcke

- Forschung & Wissenschaft
  - Die Herausgabe der Fachzeitschrift „Neuropsychiatrie“, die den Mitgliedern in analoger und digitaler Form zur Verfügung gestellt wird.
  - Die Vergabe des Wissenschaftspreises der ÖGKJP und des Ernst-Berger-Förderpreises für sozialpsychiatrische Forschung.
  - Die Bearbeitung von Agenden des Sonderfaches „Kinder und Jugendpsychiatrie und Psychotherapeutische Medizin“ (KJPP) in Kommissionen (Prüfungskommission, Ausbildungskommission, Evaluierungs- und Qualitätssicherungskommission).
  - Die Bearbeitung von KJPP-relevanten Fragestellungen und Problemlagen entlang unterschiedlicher Themenfelder in dafür eingerichteten Arbeitsgruppen (AG) (z. B. AG Kinder- und Jugendforensik, Sucht, etc.).
- Veranstaltungen & Kooperationspartnerschaften
  - Die Konzeption und Organisation des ÖGKJP-Kongresses im Zweijahresrhythmus.
  - Finanzielle Vergünstigungen für Mitglieder bei ausgewählten Veranstaltungen.
  - Das kontinuierliche Engagement in nationalen und internationalen Partnerschaften (z. B. European Society for Child and Adolescent Psychiatry (ESCAP), European Union of Medical Specialists (UEMS), Politische Kindermedizin, Suizidprävention Austria (SUPRA)).
- ÖGKJP-Akademie
  - Das Ausbildungscurriculum für Psychotherapeutische Medizin „CuPsy“.
  - Die Assistent*innentage im Rahmen der Facharzt/Fachärztinnenausbildung.
  - Die Vorbereitung und Begleitung der Facharzt/Fachärztinnenprüfungen.
  - Die Konzeption und Organisation der jährlich stattfindenden Facharztwerkstatt in Gösing.
- Repräsentanz des Sonderfaches auf politischer Ebene
  - Die Vertretung der Standesinteressen der österreichischen Kinder- und Jugendpsychiatrie in Kooperation mit den Fachgruppenvertreter*innen in den Ärztekammern sowie auf Bundesebene (z. B. Repräsentanz im Beirat Psychische Gesundheit/ BMSGPK), im Obersten Sanitätsrat, in der UbG Kommission/BMSGPK, etc.
- Homepage, Intranet & Sekretariat
  - Die ÖGKJP-Homepage https://oegkjp.at.
  - Der interne Mitgliederbereich auf der Plattform Moodle https://campus.oegkjp.at.
  - Das seit dem Frühjahr 2017 eingerichtete Sekretariat als Struktur – unter anderem für die Mitgliederverwaltung, Homepagebespielung, Organisation und Administration von Veranstaltungen.

Abgeschlossen wurde die Befragung mit zwei offenen Fragen zu Themen/Themenblöcken, die von der ÖGKJP künftig aufgegriffen und bearbeitet werden sollen.

## Ergebnisse

**Figure 1:**
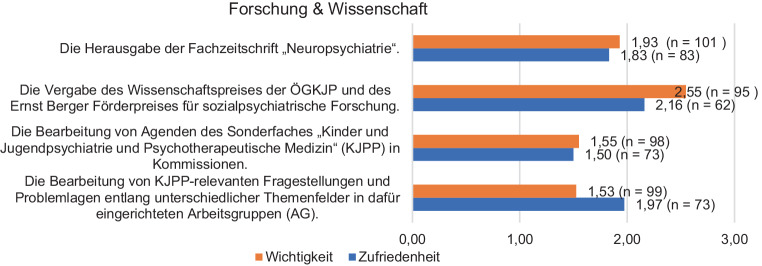

**Figure 2:**
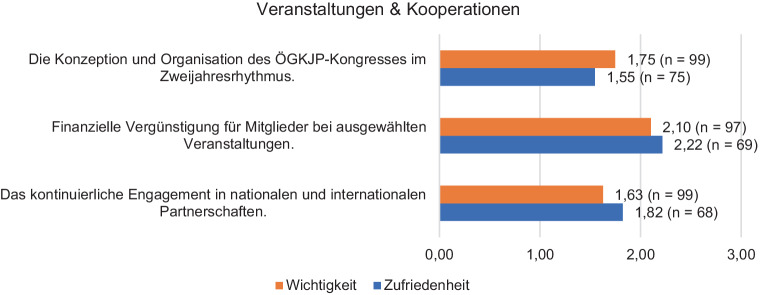

In Bezug auf den Themenblock *Forschung und Wissenschaft* bewerteten die Mitglieder die Wichtigkeit im Durchschnitt zwischen 1,53–2,55 und die Zufriedenheit mit der Umsetzung zwischen 1,50 und 2,16 (Details in Abb. 1). Hinsichtlich des Themenkomplexes Veranstaltungen und Kooperationspartnerschaften (Details siehe Abb. 2) liegt die Wichtigkeit zwischen 1,63 und 2,10, die Zufriedenheit mit der Umsetzung zwischen 1,55 und 2,22.

Die *Repräsentanz des Faches in der Öffentlichkeit* wurde als höchst wichtig erachtet (1,23), die Zufriedenheit der Mitglieder in Bezug auf die Repräsentanz des Faches in der Öffentlichkeit liegt bei 2,24 Punkten.

Die *Neuerungen im Servicebereich der Gesellschaft* wurden hinsichtlich ihrer Wichtigkeit wie folgt beurteilt: Homepage 1,82; Lernplattform Moodle 2,28 und das neue Sekretariat mit 1,83. Die Zufriedenheit der Mitglieder mit diesen Neuerungen bekommt jeweils die Note 2,22; 2,50 und 1,84.

**Figure 3:**
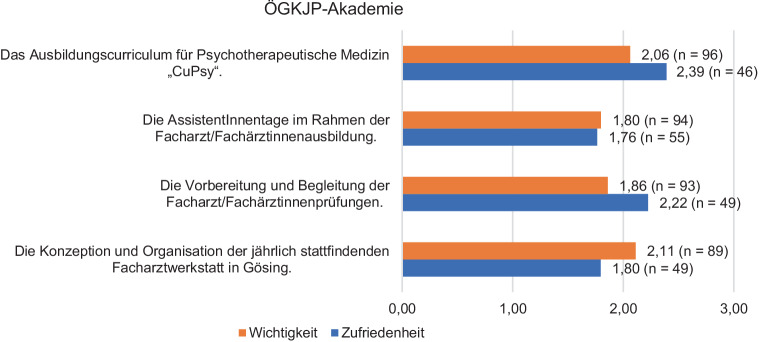

Mehr als die Hälfte der Antwortgeber*innen (ca. 54 %) erachten die *Repräsentanz des Faches in der gesellschaftlichen und politischen Öffentlichkeit von Seiten der Fachgesellschaft *als wichtiges Zukunftsthema. Über 36 % der Antwortgeber*innen erachten zudem das künftige Engagement der Fachgesellschaft bei Veranstaltungen und die Kooperationen als wichtig. Die übrigen Beurteilungen finden sich in Abb. 3.

Die Empfehlungen werden in der Form interpretiert, dass das gegenwärtige Service- und Leistungsangebot grundsätzlich als zufriedenstellend erachtet wird und die Mitglieder mit der Form der Ausführung der Services/Leistungen zufrieden sind.

Nichtsdestotrotz besteht die Absicht – und ist aus Sicht der Ergebnisse nötig – ausgewählte Themen künftig (noch) intensiver bearbeiten zu wollen – auf Basis der erteilten Anregungen dazu von Seiten der Mitglieder sowie im Einklang mit der strategischen Ausrichtung der ÖGKJP, die von Seiten des Vorstandes entlang von Leitideen kontinuierlich angepasst wird.

**Figure 4:**
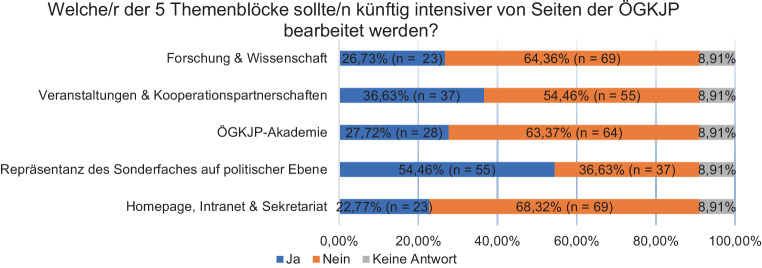

Die neue ÖGKJP-Akademie wurde hinsichtlich der Wichtigkeit zwischen 1,80 und 2,11 bewertet und in Bezug auf die Zufriedenheit mit ihrer Umsetzung mit 1,76–2,39 (Details siehe Abb. 4).

In Tab. 1 finden sich ergänzende inhaltliche Vorschläge, die von Seiten der befragten Mitglieder entlang von fünf Themenblöcken erteilt wurden. Die eigenen Themenvorschläge und allgemeinen Anregungen, zu deren Einbringung die Mitglieder ebenfalls eingeladen wurden, hatten zum Teil einen sehr konkreten Charakter. Etwa wurde vorgeschlagen, eine interne Ethikkommission einzurichten – einem Anliegen, dem Rechnung getragen wurde. Ein Ethikbeirat wurde bereits Ende 2019 eingerichtet. Angeregt wurde zudem, die transdisziplinäre Kooperation, ein wesentlicher Dreh- und Angelpunkt in der Kinder- und Jugendpsychiatrie, weiter zu stärken.

**Table 1:** 

(1) Forschung & Wissenschaft	Fokus auf sich neu entwickelnde Themen bzw. im KJP-Alltag konstant relevante Themen wie z. B. Transgender, Trauma, Autismus, Mental-health-Rehabilitation, Psychose, Trauma, Psychopharmakotherapie, Jugendforensik, Sucht, Kleinkinder, Eltern-Kind-Therapien, psychotherapeutische Medizin, Sozialpsychiatrie und Ausbildungsqualität
(2) Veranstaltungen & Kooperationspartnerschaften	Organisation von Inputs und Austauschmöglichkeiten (national/international) entlang obiger Themen
	*Organisation von Austauschmöglichkeiten mit/für folgende/n Stakeholder/n* *:* Mit Nachbarfächern, mit Pädagog*innen, für die Gruppe der niedergelassenen Fachärzt*innen
(3) ÖGKJP-Akademie	Psychotherapieausbildung und Integration von anderen Ausbildungswegen in die alte Ausbildungsordnung
	Infos und Angebote von Veranstaltungen für eine psychotherapeutische Ausbildung im Rahmen der alten Studienordnung
	Unterstützung von Kolleg*innen, die eine Psychotherapieausbildung nach dem Psychotherapiegesetz anstreben und Erteilung von Kooperationsangeboten an Psychotherapie-Ausbildungsvereine
	Prüfungsvorbereitung FA-Prüfung
	Mehr einschlägige Fortbildungsveranstaltungen (z. B. auch gezielte Schulung der Auszubildenden im Umgang mit Tabuthemen – wie etwa Gewalt innerhalb und außerhalb der Familien)
(4) Repräsentanz des Sonderfaches auf politischer Ebene	Neue oder verbesserte Rahmenbedingungen für Ausbildungsstellen
	*Mögliche Themen von Stellungnahmen in Medien:* Versorgungsprobleme und -erfordernisse Psychosoziale Problemfelder im gesamtgesellschaftlichen Kontext (z. B. Notwendigkeit niederschwelliger Zugänge für von Misshandlung und Missbrauch betroffener Kinder.)
	*Kontakt zur Politik und Standesvertretung:* Regelmäßige Thematisierung der mit dem Status als Mangelfach verbundene Problematiken (z. B. Erhöhung von Ausbildungs- und Kassenstellen)
	*Kontakt zu Bildungseinrichtungen (z.* *B. Stadtschulrat):* Verbesserung der Abstimmung in Bezug auf junge Menschen mit psychischen Erkrankungen sowie Sensibilisierung in Bezug auf den Umgang mit Betroffenen
	*AG Recht/Agenden:* Kommentieren und fachliche Einflussnahme auf (Familien-)rechtsprechung, gutachterliche QS, gesetzliche Grundlagen (Fürsorgerecht statt nur UbG), seelische Behinderung und Entwicklungsgefährdung als Rechtsgut ins KJHG, Einklagbare Eingliederungs- und Teilhabe-Unterstützung für anspruchsberechtigte Minderjährige, etc
(5) Homepage, Intranet & Sekretariat	Relaunch Website

Ebenfalls deutlich aus den Anregungen hervorgegangen sind die zentralen Spannungsfelder, die im Sonderfach seit langem bestehen: hierzu zählt vor allem die kinder- und jugendpsychiatrische Mangelversorgung, und die damit in Verbindung stehenden Belastungen der Fachärzt*innen für Kinder- und Jugendpsychiatrie und in benachbarten Berufsfeldern Tätigen.

## Diskussion

Non-Profit-Organisationen leben in einem Dilemma zwischen Ehrenamtlichkeit und Professionalität, was per se kein Widerspruch sein muss. Allerdings wird gerade bei wachsenden Organisationen die Diskrepanz zwischen bezahlter Arbeit und Ehrenamtlichkeit und Leistungsfähigkeit sehr deutlich.

**Figure 5:**
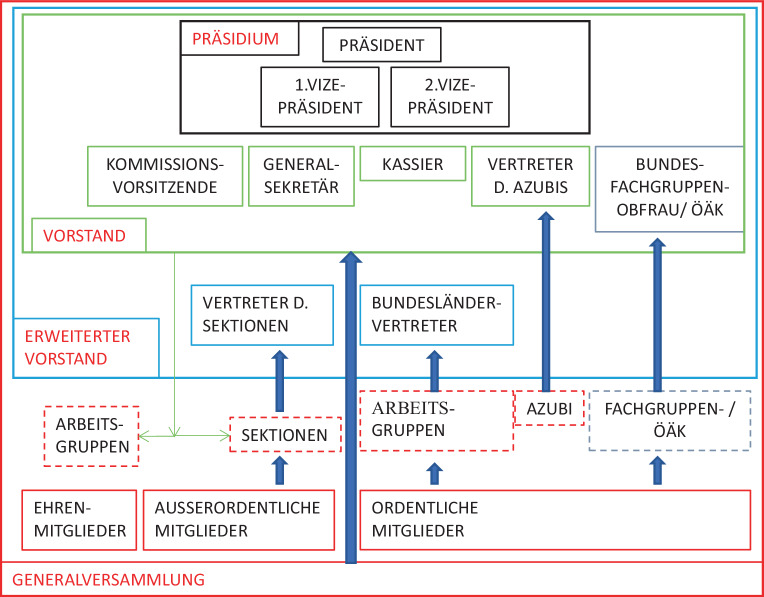

Der Mitgliederstand unserer Fachgesellschaft hat in den letzten Jahren deutlich zugenommen, gleichzeitig sind auch die Aufgaben differenzierter und mengenmäßig mehr geworden. Die Arbeit unserer Fachgesellschaft geschieht in erster Linie in den Kommissionen, Arbeitsgruppen, Landesgruppen, Sektionen und im Vorstand (siehe auch Abb. 5). Die Ausbildungskommission ist einerseits für die curriculare Ausgestaltung der Ausbildung zuständig, insbesondere seit der Ärzteausbildungsordnung 2015 [6] für die Ausgestaltung des CuPsy (Curriculum Psychotherapeutische Medizin), sie ist auch der Kopf der ÖGKJP-Akademie. Die Prüfungskommission erarbeitet die Facharztprüfungsinhalte und führt diese im Auftrag der ÖÄK gemeinsam mit dieser durch. Die dritte Kommission – Evaluations und Qualitätssicherung – hat ein komplexes Aufgabengebiet und arbeitet höchst eigenverantwortlich an qualitätssichernden Themen und dabei sehr eng mit dem Vorstand und im Auftrag des Vorstandes. Momentan gibt es 9 Arbeitsgruppen (https://oegkjp.at/arbeitsgruppen). Im Durchschnitt arbeiten in den Kommissionen und in den AGs in etwa 5–20 Mitglieder aktiv mit. Die Sektionen sind die Organisationsform für alle *allied professions* wie Pflege, (Klinische) Pädagog*innen, Psycholog*innen oder Soziale Arbeit.

Nach der Erarbeitung der neuen Strategie lag es nahe – im Sinne einer *Lernenden Organisation* die Mitglieder zu befragen, inwieweit die Veränderungen von diesen als wichtig wahrgenommen werden und wie zufrieden sie damit sind. Die Ergebnisse zeigen, dass die Mitglieder die vom Vorstand gesetzten Ziele teilen und wichtig finden und die Mitglieder auch mit der Umsetzung recht zufrieden sind.

Bei den Empfehlungen für zukünftige Aktivitäten der Fachgesellschaft werden zwei Themen wesentlich hervorgehoben: *Veranstaltungen und Kooperationspartnerschaften* wie das Thema der *Repräsentanz des Sonderfaches auf politischer Ebene*. Insbesondere zu letzterem Thema gab es und gibt es verschiedene Aktivitäten. Einerseits wurde ein Konzept für die Öffentlichkeitsarbeit entwickelt und mit dem Abonnement bei der APA (Austrian Presse Agentur) eine Reihe von Stellungnahmen veröffentlicht [7]. Weiters wurde versucht, die politischen Kontakte zu verstärken und unsere Anliegen der Politik vorzutragen, auf Bundesebene gab es dazu einen Termin im Justizministerium und einen Termin mit Bundesminister Rudolf Anschober. Die Reaktionen waren durchaus positiv und lassen hoffen, dass unsere Forderungen und Vorschläge Gehör finden.

Im Bereich der Veranstaltungen ist die Fachgesellschaft sehr aktiv und diese werden offenbar auch gut angenommen. Hier wird der Ausbau der ÖGKJP-Akademie im Zentrum der weiteren Entwicklung stehen, eine strukturelle Verstärkung durch 10 h Sekretariat wurde heuer bereits umgesetzt.

Die Ergebnisse der Mitgliederbefragung haben den Vorstand einerseits bestärkt und andererseits auf die besonderen Wünsche der Mitglieder hingewiesen. Die Umsetzung dieser Ideen und das Management einer NPO bei dieser Thematik und in dieser Organisationsform sind als zirkuläre Prozesse zu verstehen, die sich in Rückkoppelung interner Vorstellungen, selbstgestellter Aufträge, Aufträge aus den gesetzlichen Vorgaben und der Realität der Kinder und Jugendpsychiatrie in Österreich generieren. Kühl [8] beschreibt Management idealerweise als einen kontinuierlichen Prozess einer ununterbrochenen Zielvorstellung und der Erwartung diese Ziele auch zu erreichen mit dem gleichzeitigen Wissen, das Ziel nie erreichen zu können. Krejci [9] formuliert das so: weniger konkrete Zielvorgaben, eher großzügige Zielformulierungen, Planung und Entwicklung sind zirkuläre Prozesse und kein lineares Vorgehen, diese Zyklen sollen überschau- und planbar bleiben, keinen rigiden Planungsprozessen folgen, sondern die Potenziale jener Menschen nutzen, die vorhanden und aktiv sind.
